# Kinetics of Carbon Nanotubes and Graphene Growth on Iron and Steel: Evidencing the Mechanisms of Carbon Formation

**DOI:** 10.3390/nano11010143

**Published:** 2021-01-08

**Authors:** Luís Sousa Lobo, Sónia A. C. Carabineiro

**Affiliations:** LAQV-REQUIMTE, Department of Chemistry, NOVA School of Science and Technology, Universidade NOVA de Lisboa, 2829-516 Caparica, Portugal; sonia.carabineiro@fct.unl.pt

**Keywords:** CNTs growth, Fe-steel catalysis, kinetics vs. mechanism, combined metal catalysis, metal nanoparticles

## Abstract

Carbon formation on steel has recently become an active research area with several important applications, using either carbon nanotubes (CNTs) or graphene structures. The production of vertically aligned CNT (VACNT) forests with combined metals has been explored with important results. Detailed kinetics is the best approach to understand a mechanism. The growth behavior seems complex but can be simplified through the knowledge of the three more common alternative reaction mechanisms/routes. The time required to optimize the production and properties might be reduced. The mechanistic proposal reported in 1971 was better explained recently. The volcano shape Arrhenius plot reported is observed only when Fe, Co, and Ni are used as reaction catalysts. Other metals are catalytically active at higher temperatures, following a different route, which does not require surface catalysis decomposition of the reactive gas. C_2_H_2_ and low olefins react well, but CH_4_ is not reactive via this surface catalysis route. Optimizing production of CNTs, research work is usually based on previous experience, but solid-state science-based studies are available.

## 1. Introduction

Carbon formation has become an active research area, particularly since 1990. Ni and Co are of particular interest up to 600 °C and Fe up to 650 °C, because they are active in surface catalysis of C_2_H_2_ and low olefins, their (111) surfaces being the most active above those temperatures. The route operating below the temperature of surface catalysis is the same as at higher temperatures, but an alternative carbon growth route (“alternative mechanism”) operates with most transition metals. In this case, the role of the metal is just to allow interstitial carbon atoms diffusion, dissolving in the side of the nanoparticles and nucleating and growing CNTs on the opposite side. Many transition metals are operative at nano level, to activate CNTs growth, providing that pyrolytic formed C_2_, C_3_ species impinge on the catalyst surface. Research about C formation, occurring since 1930, opened the way to this fascinating area of science and technology.

Palmer and Cullis reviewed carbon formation from gases in 1965 [1] and included a transmission electron microscopy (TEM) image of carbon filaments grown on steel, from the work of Singer and Grummer, using a propane-air flame, published earlier, in 1959 [2]. Palmer and Cullis discussed kinetics and mechanism theories in some detail, listing the following theories: (1) The C_2_ theory; (2) the atomic carbon theory; (3) the C_3_ theory; (4) the acetylene theory; (5) hydrocarbon polymerization theories; (6) the surface decomposition theory; (7) the Boudouard reaction theory, mentioning the role of butadiene as an important intermediate. Palmer and Cullis also offered suggestions for future work and stated: “There may have been too much emphasis in the past upon searching for the mechanism of carbon formation, as though it was unique”.

**Figure 1:**
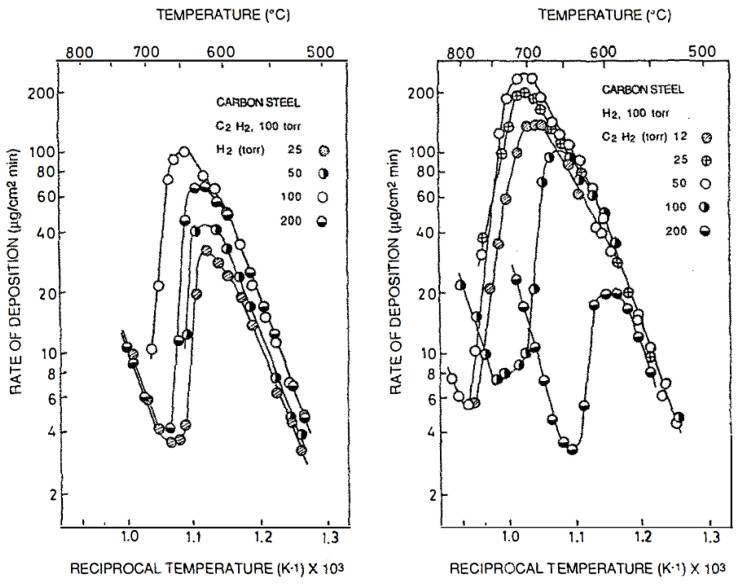
Arrhenius plots of the rates of carbon formation observed on steel from C_2_H_2_. (check also Figure 2). Reprinted from [9], with permission of Elsevier. Every point in the plot corresponds to a steady-state carbon deposition rate register (linearity) in a microbalance. This kinetic behavior was observed first on Ni in 1971 [7].

Nowadays, there is a lot of information on the formation of specific types of carbon materials, namely carbon nanotubes (CNTs) and graphene layers and the technologic applications of these materials is very wide and increasing every year. However, the growth mechanism of carbon growth is still not well understood [3,4]. The case of Fe and steel has specific aspects and slower kinetics (check Table 1). The kinetics and alternative mechanisms of carbon formation have been recently revised by Lobo (check Figure 1) [5]. The carbide Fe_3_C is stable in contact with carbon. Detailed reviews on studies of carbon formation from gases in the period 1930–1965 are available, which are very useful to get information on the experimental behavior observed and on possible mechanisms operating [1,2,6]. Jost’s book gives theoretical and experimental information on diffusion in membranes and through metals [6].

The kinetic studies performed by Lobo and Trimm in 1971/72 on carbon formation from olefins and acetylene catalyzed by transition metals gave detailed kinetic data, evidencing the alternative operating mechanisms [7,8]. The rates of catalytic carbon formation from acetylene and low olefins were found to be faster on Ni, slower on Co, and much slower on Fe and steel (check Table 1). Kinetic studies measure the kinetics of C formation. The much lower growth rate of CNTs on Fe may be due to slower C bulk diffusion or larger nanoparticles (longer C diffusion distances required).

**Figure 2:**
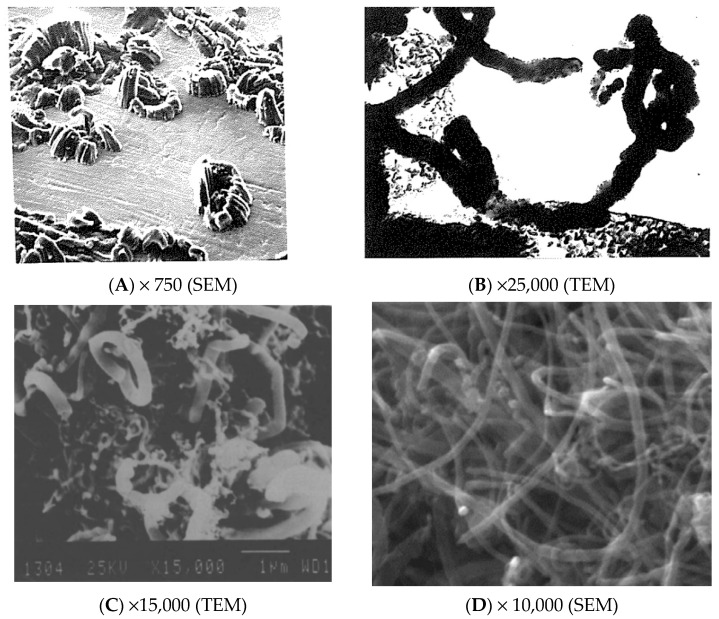
SEM/TEM Images of CNTs grown on Fe (**A**,**B**) [45], on steel (**C**) [9], and on AISI 316L (**D**) [36]. Reprinted from [9,36] (with permission of Elsevier) and from [45] (open PhD text).

Association of kinetics with images (TEM, SEM) was essential to discover the alternative mechanisms operating (check Figure 2). However, detailed kinetic studies of carbon formation are rare nowadays. That knowledge is of great interest to optimize production and CNTs and graphene properties more easily. The rates of individual CNTs depend on geometry and on the rate determining step operating. The overall rate is more consistent to confirm the mechanism taking place. Lobo and Trimm studied kinetics under steady-state carbon formation at different temperatures to evaluate the activation energies (E_a_), and under different pressures to evaluate reaction orders [7,8].

## 2. Recent Experimental Work

Jourdain and Bichara published, in 2016, an excellent and comprehensive review on the growth of carbon nanotubes (CNTs), covering mostly thermodynamics [11]. However, a better study on kinetics will help to understand the alternative mechanisms and optimize production. Many studies on carbon formation on steel and iron/Fe_3_C are available. A selection is listed in Table 2. The most used gas is acetylene and the temperature is usually above 650 °C.

Those studies test different temperatures, gases, pressures, and pre-treatment modes checking the properties of CNTs structures and densities obtained. The properties optimization and production rates of CNTs will be facilitated when the mechanism operating is well understood. Detailed knowledge of kinetics is the key to more easily optimize the growth process.

## 3. Kinetic Routes/Mechanisms of CNTs and Graphene Formation

**Figure 3:**
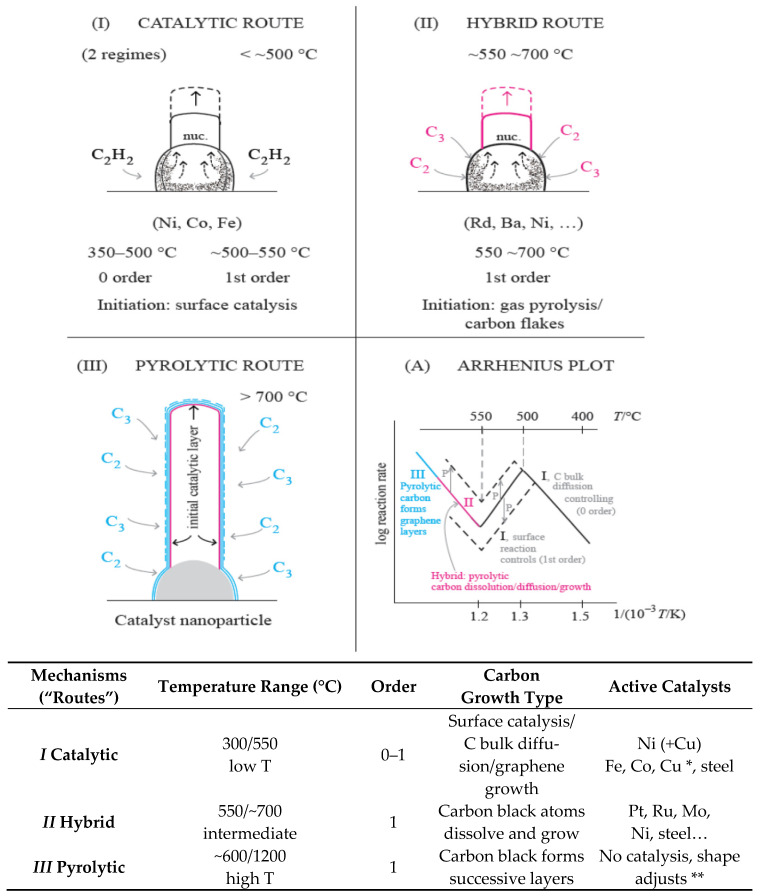
Explanation of the three alternative catalytic mechanisms/routes of carbon formation from hydrocarbons over different temperature/pressure ranges. When more than one mechanism may operate, the faster one prevails (adapted from [51] with permission from MDPI). * With Cu: at 250 °C. ** Graphene functionalization properties are extensively studied today.

Epitaxial growth on solid surfaces has been studied in some detail. Three growth modes are known: Island, layer-plus-island, and layer-by-layer [46]. However, nucleation of graphene on Ni, Fe, and Cu at low temperatures (300–550 °C) occurs through C atoms bulk diffusion supplied by gas decomposition (Mechanism/Route I) or carbon black C atoms (Mechanism/Route II), that dissolve and keep their individuality, moving interstitially in the metal catalyst. Layer-by-layer growth occurs only in the pyrolytic route (Figure 3). The Tammann temperature of Cu (406 °C) is much lower than Ni, Co, and Fe [47]. Route I operates with Fe, Co, Ni, and Cu only, using C_2_H_2_ or low olefins [5].

As mentioned above, detailed kinetics is the best approach to prove which mechanism is operating. The detailed research work on diffusion of atoms in and through solids published from 1920 to 1960 should be used, particularly to get data on H, C, N, and O bulk diffusion in transition and noble metals [48,49]. Diffusion in solids is a complex process, but diffusion of C, N, and O in transition metals is interstitial: The atomic radius of solute C and solvent (transition metal) is below 0.59, the usual condition for that type of diffusion to occur [48,49].

It is essential to perform the experimental work under steady-state deposition rates (initial solid-state adjustments have finished). When the flux is constant, the first Fick’s law applies:

J = −D dC/dx

where J is the flux and dC/dx is the concentration gradient. Two different phases may operate in the catalyst nanoparticle (gas side vs. CNT growth side), adjusting the solid-state geometry to a C flux steady-state operating regime [50].

The Arrhenius plots shown in Figure 1 evidence the change of rate determining step at about 700 °C. Explanation of that change has been recently reminded [51]. Every point in the Arrhenius plot is a register of a steady-state deposition rate experiment (kinetic linearity observed) [5], that is, a full steady state C formation experiment, lasting 2 or 3 h. The volcano shape maximum with Fe and steel occurs at higher temperatures than with Ni and Co. This is due to the lower rate of the 1st reaction step (catalytic surface reaction), so that the prevalence of a lower 2nd step (C bulk diffusion through the solid catalyst) only occurs at higher temperatures. The dependence of temperature of the reaction rate observed in the lower temperature side of the volcano plot with Ni was ~33 kcal/mole. This is the activation energy of C atoms bulk diffusion in Ni. That value and zero order gas pressure dependence of the C formation reaction rate were regarded as proof of the mechanism operating, as reported in 1971 [7,8,45]. Baker et al. described in detail the TEM in-situ growth geometry of CNTs (no kinetic experiments) [52] and followed the mechanism proposed by Lobo and Trimm based in 160 kinetic experiments, using a CI Electronics microbalance adapted to automatically change ranges in long experiments, if required [7,8]. In 2011, Lobo, Figueiredo, and Bernardo summarized their approach to the mechanism in the early 1970s [53].

A successful kinetic study requires an initial transition: The change of weight is fast in the first few minutes and then decreases to a sustained rate (kinetic linearity, as mentioned above). Our experiments with iron and steel were initiated at a lower temperature (ex. 500 °C) for 2 h. After that the temperature was increased by stages (ex. 15 °C) allowing enough time at each stage to confirm a steady state operating (straight line in the weight register). The initial transition may include phase changes of the nanoparticle catalyst bulk [50]. At lower temperatures, the prevailing bulk phase with Ni is the metal itself, but with iron it is Fe_3_C. Latorre et al. proposed a phenomenological kinetic model and discussed the nucleation and growth of CNTs in some detail [54].

Ermakova et al. [55] studied carbon formation from CH_4_/H_2_ using Fe on various supports: SiO_2_, Al_2_O_3_, and ZrO_2_ in the range 650–800 °C. The maximal carbon yield was obtained with SiO_2_. Metal filled carbon tubes were frequently filled with Fe particles and commented: “That can be hardly explained unless the quasi-liquid state of the metal is assumed”, and concluded that a high fluidity of iron-carbon particles was observed above 640 °C. However, the explanation is the sintering-like behavior of the nanoparticles due to contact interaction [56]. The sintering temperature of Fe is 632 °C.

Puretzky et al. [14,57] studied the kinetic CNTs’ growth using acetylene/Ar/H_2_/in the range 550–900 °C. These authors used multilayer metal films of 10nm Al and Fe or Mo as catalysts and a flow of C_2_H_2_ (6 sccm) diluted in Ar (ex: 2000 sccm) and H_2_ (ex: 400 sccm) [14]. The reason for this gas dilution can be understood by our recent analysis of high temperature carbon formation kinetics [58]. Low hydrocarbon partial pressure is the key to keep Route II operating at higher temperatures with a faster rate and avoiding pyrolytic graphene layers deposition (Route III, pyrolytic). In that study, a volcano shape of the Arrhenius plots of the rates vs. temperature (check ref. [14], Figures 13, 21, and 22), but the orders of reaction were not evaluated. In our studies, the orders of reaction were always evaluated experimentally. With that information, the alternative mechanisms operating were more easily distinguished. The two sides of the volcano correspond to the same mechanism (Route I, Catalytic), but with a change of the rate-determining step from C bulk diffusion to surface reaction decomposition of the gas reactant (C_2_H_2_ and low olefins, only). The other C formation gases only operate at higher temperatures by impingement of pyrolytic formed carbon (C_2_,C_3_…), C atoms entering the bulk of the catalyst and growing catalytically on the other side of the nano-particle [51]. In the studies of Puretzky et al., the reason a volcano shape was observed in the plot of the variation of the growth rates as a function of temperature was attributed to acetylene flow rates [57]. This is not correct. They did not measure reaction orders (alternative gas pressure steady-state experiments). The reaction order changes from zero (temperatures below the volcano maximum) to one (at temperatures above the maximum) [7,8]. This is the reason for the volcano shape observed.

The activity of Cu has been studied in detail by Shaikjee et al. using C_2_H_2_ at 195 °C and 250 °C [59]. The Tammann temperature of Cu is 405 °C. The absence of data on CNTs formation using Cu via Route II may be related to the stability of the nanoparticles shape.

Overall knowledge of the CNTs and graphene alternative growth mechanisms [5,8,58] is important to optimize rate, structure, and desired properties. Route I operates with acetylene and C_2_ to C_4_ olefins and CO. At higher temperatures, CNTs can be formed by route II only.

Studies on C formation on Ni, Co, and Fe in the 70′s were mainly performed to minimize the problems in the steam-reforming industry. Ni-Cu catalysts were used to reduce the problem, but still the need to stop the production from time to time due to catalyst deactivation by carbon formation was costly. CNTs were observed to grow easily from transition metals [7,35], but their properties were not known at the time. Carneiro, Baker, and co-authors studied CNTs’ growth on Fe-Ni and Fe-Cu from CO/H_2_ at ~700 °C [13,25]. They studied the structure of the CNTs formed. No kinetic studies were reported. An update of the observed kinetics of CNTs growth was published by Lobo [5].

The wider use of CNTs for many purposes and industrial production started after the work of Iijima in 1991 [60]. Single layer CNTs were produced in 1993 by Iijima [61] and Bethune [62].

Roumeli recently published a study of vertically aligned CNT forests grown on stainless steel surfaces, including adhesion tests between the tubes and the steel substrate to test their adhesion performance using 4 types of steels [42].

Concerning graphene, the deposition of layers at high temperatures is a transition from the CNTs growth by the hybrid route to the pyrolytic route, but the deposition rate observed follows the same Arrhenius plot line [58]: Almost a paradox (check Figure 3, (A)). This must be understood—it is a change of mechanism with a continuous line in the Arrhenius plot: At lower temperatures, C_2_/C_3_ rate of deposition controls the rate; at higher temperatures, C_2_/C_3_ rate of deposition dominates and covers the catalyst surface with graphene layers–pyrolytic route.

Koyama and Katsuki et al. produced carbon fibers via pyrolysis of benzene and naphthalene at temperatures above 1000 °C in 1972 [63,64]. Tibbetts reported the production of carbon fibers by pyrolysis of CH_4_ in stainless steel tubes in the range 950–1075 °C [65]. Figueiredo and co-workers studied carbon formation from CH_4_ using Fe-Ni, Fe-Co, and Ni-Co in the range of temperatures 650–950 °C [66] and using Fe-Mo, in the range of temperatures 500–800 °C [67]. The carbon formation reaction using methane does not operate by the catalytic route. Only via gas pyrolysis and the hybrid route the formation of CNTs is possible. High temperatures are required.

## 4. Role of Solid-State Chemistry in CNTs Growth Mechanism

Boyes et al. discussed very recently studies using environmental TEM of single atom dynamics in chemical reactions, including Pt/C, Cu, and Co catalyst nanoparticles [68]. No reference to solid-state chemistry was included in the discussion. A wrong understanding of the mechanism operating is very common nowadays. Solid-state chemistry became an important area of science and technology in the period 1910–1980 [6,44,69,70,71], but is frequently ignored nowadays. However, we need solid-state chemistry knowledge to understand the CNTs growth mechanism.

Diffusion of C, N, and O atoms in transition metals is interstitial, due to the ratio of the covalent radius of the metal solvent and dissolved atoms (AR_sol_/AR_solv_) being less than 0.60 (0.50 for Fe) [51,58]. The solid-state phases operating during CNT growth are mostly Ni and a Ni_3_C layer at the gas phase reaction side. Diffusion in solids (D) is a process with activation energy (Ea) dependence from temperature (T) [50]:

D = A e ^−Ea/RT^

where A is the pre-exponential factor and R is the ideal gas constant.

**Figure 4:**
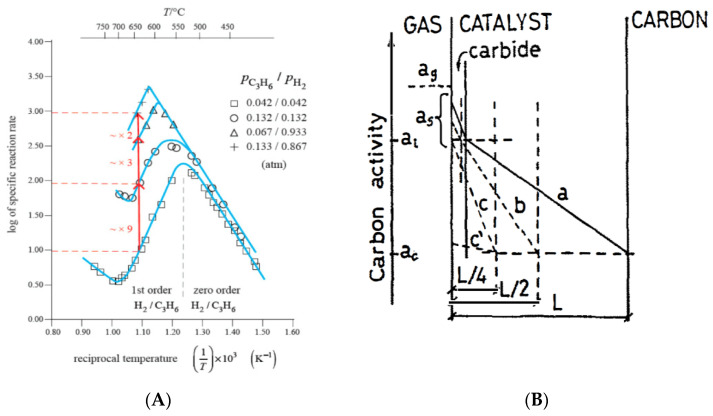
(**A**) Experimental results measured with a vacuum microbalance (Arrhenius plot) proving a C bulk diffusion mechanism and the change or rate determining step at 550–600 °C. Reprinted from [51] with permission from MDPI. (**B**) Carbon bulk diffusion through the catalyst bulk during CNT growth: Carbon concentration/activity profiles through the catalyst for three different diffusion paths. The phase thicknesses are stable during reaction. With Ni, the metal is the main phase during CNTs formation, but with Fe and steel, the main stable phase with graphite is a carbide (usually Fe_3_C). Reprinted from [9] with permission from Elsevier.

Reaction rate control is sometimes assumed when an apparent exponential dependence of the rate with temperature is observed. An Arrhenius plot is required. A zero order reaction is a good indication of C bulk diffusion control. Linearity observed in a weight vs. time register indicates that a steady-state C formation mechanism is operating [5]. With Ni, evidence of a change of rate determining step at ~550–600 °C from C bulk diffusion to catalytic surface reaction producing C atoms is very obvious from the kinetics register (Figure 4A). Budnikov and Ginstling discussed the kinetics observed in solid-state chemistry in some detail in their book (chapter 5) [70]. Lobo and Franco observed the kinetic behavior of carbon formation using various steels [9].

A scheme of the stable phases during CNTs formation on Ni (above ~300 °C) is shown for three different thicknesses in Figure 4B. The stable carbide phase is Ni_3_C in this case.

However, it is different with iron, because Fe_3_C is metastable in the temperature range of CNTs formation. However, its Tammann temperature is 632 °C [47]. This explains the coalescence of the catalyst nanoparticles observed when the hydrocarbon pressure is low (check Figure 4). Interstitial C atoms diffusion through Fe, Co, Ni, and Fe_3_C is easy.

The transition from Route I to Route II was not considered. However, to understand which mechanism is operating, kinetics is the key: In parallel reactions routes, the faster route prevails.

Robertson and co-workers used Fe as the main catalyst in several studies in 2007–2014 [17,18,26,30,72,73,74,75]. They compared Fe with Cu in CNTs forest growth, and tested Fe-Ta co-catalysts. They discussed CNT growth emphasizing the adsorption step, the carbide heat of formation, and carbon solubility [74].

Nessim et al. [76] studied alternative preheating modes and geometry in hot-wall and cold-wall flow reactors, in carbon nanotubes formation on Fe/SiO_2_ in the temperature range 730–770 °C using C_2_H_4_/H_2_. Nessim also published in 2010 a review article on properties, synthesis, and growth mechanisms of CNTs (focus in CVD) using Fe, Ni, and Pt [77]. No distinction between the two different mechanisms operating was assumed. That distinction is relevant: More options of metal catalysts and C containing gases in the hybrid route, pyrolytically initiated in one side and solid-state kinetics operating on the other side [78,79,80].

Nucleation of the initial base or top of the catalyst nanoparticle graphene nucleus is formed, which grows and bends at the edges of a nano-crystal face of the nanoparticle more active in initial graphene nucleation. This mechanism was studied in detail by Garcia-Lekue et al. [81].

A detailed study of Gao et al. using C_2_H_2_ (10 sccm) and H_2_ (490 sccm) at 600 °C showed that, at higher temperatures, Fe nanoparticles tend to grow in size, reducing the CNT growth rate [82]. Near or above the Tammann temperature (T_Ta_), sintering like changes tend to occur [56]. The T_Ta_ at nanoscale is slightly lower. Panahi et al. tested SS-304, SS-316, and SS-316L with alternative pre-treatment methods. Stainless steel SS-316 gave the best effectiveness in promoting CNTs growth [43].

At much lower temperatures, other iron carbides are the stable phases: In Fisher–Tropsch processes (150–320 °C), the active Fe phases are Fe_2.2_ and Fe_5_C_2_. At 360 °C, Fe_5_C_2_ turns to Fe_3_C.

Some authors mention low solubility as an indication of slower diffusivity of C in a metal catalyst. However, experimental data shows that high solubility corresponds to low diffusivity: The interstitial routes for diffusion are blocked. An introduction to solid-state diffusion and alternative diffusion types is well summarized by Schmalzried [71]: Vacancy diffusion, interstitial diffusion, interstitialcy diffusion. An Arrhenius plot of interstitial diffusion of C in Fe was shown as evidence of the temperature dependence of interstitial diffusivity of atoms in solids [9,58]. The Tammann temperature of iron is 632 °C. This helps to understand the increase of size of Fe nanoparticles observed above 700 °C.

Li et al. reviewed and discussed carbon nanocoils growth [22]. Yao et al. reported CNTs formation using plastic films as a source of C for CNTs growth [83]. This is a route of great interest to reduce an environmental problem of today’s way of life, particularly present in the oceans.

Sengupta et al. studied CNTs formation and evidenced the tip-growth mechanism operating. They used propane and a thin layer of Fe (~20 nm) pretreated with hydrogen for 10 min at the reaction temperature (650–750–850–950 °C) [21].

With propane, the catalytic mechanism I does not operate, only the hybrid mechanism II. A higher temperature is required (check Figure 3). The catalytic mechanism only operates with acetylene and low olefins, which decompose catalytically on Ni (111) surfaces. Knowledge of the alternative growth mechanisms and its different kinetic behavior is an important support to optimize CNTs production and properties for alternative uses [5]. Zhong et al. studied self-termination [72].

Optimum pressures: High when Route I is operating [5], low when Route II is operating [58]. In one case, the growth rate is independent of pressure when the carbon bulk diffusion step is controlling, but the rate goes down when the surface diffusion step is controlling. To avoid that, higher pressures are required (check Figure 1 and Figure 3, (A)). In the hybrid growth route, the lower pressure and higher temperature enable this route to operate faster. Various metals and alloys, other than Fe, Co, or Ni are active as catalysts for this carbon formation route.

There are many molecular simulation based studies. This approach has shortcomings and may lead to errors in understanding the catalyzed solid-state based CNT growth processes.

## 5. CNT Forests Growth Optimization vs. Kinetics and Mechanisms

**Figure 5:**
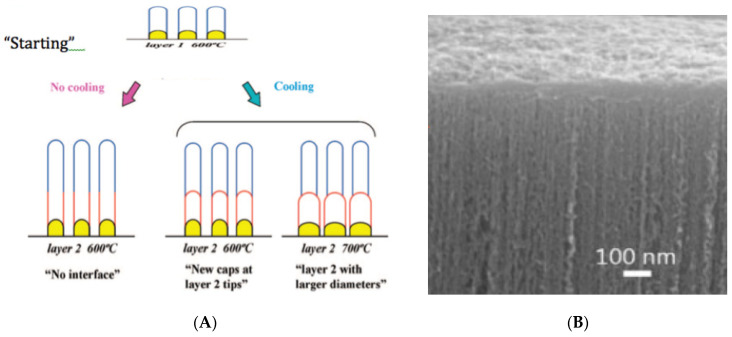
(**A**) Growth of CNT forests. Reprinted from Iwasaki et al. [18] with permission from ACS. (**B**) Closed Packed CNT Forests. Reprinted from Robertson et al. [26] with permission from Wiley.

Yamazaki et al. [84], Iwasaki et al. [18], and Robertson et al. [26,72,73,74,75] studied the growth of vertically aligned CNTs in detail (check Figure 5A,B). In this case, an extra kinetic step is present: Diffusion of the reactant gas through the thin space between the CNTs. Could that step be rate-limiting when the CNTs are very long? CNT forests are sometimes grown from CH_4_. However, in this case, mechanism I (catalytic) is not operative. Mechanism II, operating at higher temperatures, is required (check Figure 3 and Table 2). Forests of CNTs (check Figure 5B) became recently the object of growth optimization to increase production and reduce costs. Lee et al. [85], Bedewy et al. [86], Park et al. [87], Meshot et al. [88] and Yang et al. [30,75] analyzed the CNT’s forest growth, recently.

Underlayer vs. nanoparticle size, spacing, and stability during reaction have been studied by several authors [78,79,80]. Delzeit et al. studied Fe on a thick Ir underlayer, trying also Mo added to Fe. They used CO as a reacting gas and had to use 900 °C as reaction temperature [78]. In fact, mechanism I is not active with CO, and only mechanism *II* operates. With Fe, that temperature is required. Burt et al. used Fe on Al_2_O_3_ grains on Si and SiO_2_ substrates, but used ethanol as a reacting gas [79]. So only reaction Route II is operative. The reaction was performed at 800 °C with 4% H_2_. Low pressures are more effective to reach higher rates when route/mechanism II is operating. This seems a paradox, but has been explained in detail, recently [58].

A good explanation for the growth of the size of the Fe nanoparticles (“larger diameter”), causing enlargement of the CNTs diameter, shown in Figure 5A at 700 °C, is sintering-like behavior of solid-solid contacts above the Tammann temperature of Fe (632 °C), as remarked above (point 5). The rates of CNTs growth from particles with different diameter *d* are proportional to *1*/*d*^2^. The diffusion distances are proportional to *1*/*d*, and the growth perimeter is also proportional to *1*/*d*.

This proportionality helps us to understand that in a sample with various nanoparticle sizes, and so with different growth rates, the kinetic model applies: The effect of changing pressure and/or temperature applies overall in the system. The detailed study of Nessim et al. in 2008 tuning vertically aligned CNTs (VACNTs) diameter growth on Fe can be better understood considering that they operated at 770 °C, well above the T_Ta_ of Fe [89].

Baker proposed that C bulk diffusion through Ni particles is due to a temperature gradient [52,90]. We consider this to not be correct [5]. The fact that the growth rate on Ni below 550 °C is not due to a temperature gradient can also be avoided, knowing that the rates are exactly the same with C_2_, C_3_, and C_4_ olefins, and C formation from C_4_H_4_ is endothermic [5]. However, the main error is the assumption that heat, being a consequence of the reaction (exothermic), may be its cause. This infringes on the causality principle. Baker’s proposal was sustained for 20 years [90]. C bulk diffusion is due to a dissolved C concentration gradient between the two operating sides of the catalyst [7,8].

## 6. CNTs Application Areas

Thin graphene films can be formed following route/mechanism III (pyrolysis) but operating at the “border” of the required temperature and pressure conditions (slow deposition rates). Good graphene thin films have been formed by Sarno et al. [91], Romero et al. [34], and more recently by Um et al. [92]. Additionally, a book by Venables on “*Introduction to surface and thin film processes*” is available [46].

CNTs are an important basis nowadays for applications in many areas. Harris summarized those uses, covering electronic, mechanical, optical, thermal, chemical, and biology areas [3].

The studies by Treacy et al. in 1996 on the changes of mechanical properties showed that CNTs might be useful in strong, lightweight composite materials [93]. Exceptionally high young modulus were observed for individual CNTs. The very high number of citations reveals the importance of this finding. Gao et al. and Adhikary et al. recently revised the mechanical properties and microstructure of cement-based materials searching for the best structure of the CNTs to its reinforcement [82,94]. A book by Guceri and Gogotsi from an ASI NATO meeting on nanofibrous materials is available [95].

The electrical conductivity of the CNTs is important for several uses. The studies by Ebbesen et al. published in 1996 on individual electronically properties of CNTs have stimulated that study and optimization for particular applications [96]. Abrupt jumps in conductivity were observed as temperature varied. The number of citations of these articles evidences the growing use of CNTs in electronics. Increase of electrical conductivity of Fe CNT sheets. Enhancement of electrical conductivity adding Cu to Fe has been recently reported by Earp et al. [97].

CNTs have recently being tested with success in drug delivery, particularly in cancer treatment, and may progressively replace the current treatments of surgery, radiation therapy, and chemotherapy.

## 7. Conclusions

1. Detailed kinetic studies of catalytic carbon formation enabled an important scientific progress proving the modes of growth-established in 1971 for Ni, in 1980 for Co, and in 1990 for Fe and steel. The kinetics and transitions of the alternative mechanisms have recently been studied in more detail. The approach based in “rational recipes” [98] is much better than atomic scale simulations [99], which ignore experimental behavior and basic rules of solid-state chemistry.
2. The kinetic studies of CNTs formation and the knowledge of which mechanism is operating (catalytic, hybrid, or pyrolytic) saves much time in optimizing the experimental conditions and in adjusting the CNTs’ properties to a desired use.
3. Without the detailed kinetic analysis, the “main change” of rate, corresponding to the volcano-shape Arrhenius plot, is usually seen as a change of mechanism. However, it is, in fact, just a change of rate determining step. The change of mechanism from Route I to Route II is not usually understood. The restriction to Fe, Co, Ni, and C_2_H_2_ and low olefins is mandatory for Route I to operate, but Route II is operative with a C containing gas and many transition metals.
4. The rates of diffusion of C through Fe and steel are much slower than through Ni or Co. The transition of rate determining step (“volcano”) occurs at ~700 °C (Figure 1) instead of ~500 °C for that reason.
5. Additionally, the linear increase of rate from 600 °C to 1200 °C includes a major change of mechanism from Route II (hybrid) to pyrolytic external C layers deposition (Route III, no catalysis). This transition is invisible in the Arrhenius plot, just showing continuous straight-line temperature dependence.
6. The structure and properties of CNTs are easier to adjust when the particular growth mechanism operating is known, which is now the case. Iron has applications in coatings, protective layers, antifouling substrates for metallic pipelines and blades, rails, etc. Optimizing the production and properties is easier when the growth mechanism is well understood.

## Figures and Tables

**Table 1:** Comparison of the rates of carbon formation (r_w_, µg/cm^2^ min) on Ni, Co, and Fe/steel at T = 500 °C (C bulk diffusion control). Pressures: Hydrocarbon 100 torr, hydrogen 100 torr (except AISI 302 steel: Hydrogen pressure, 500 torr). E_a_, kJ/mole. Data from Lobo, Franco (1990) [9].

Gas	Kinetics	Ni	Co	Fe	Steel	AISI 302
C_2_H_2_	r_w_	85	20	2	1	-
	E_a_	130	134	88–100	180	-
C_4_H_8_	r_w_	50	15	2	1.5	0.017
	E_a_	121	142	188	171	-
Reference		[7,8]	[10]	[10]	[9]	-

**Table 2:** Selection of recent publications on catalytic carbon formation on steel or Fe/Fe_3_C. The temperatures used by the various groups are close to the maximum rates observed in Figure 1.

Year	1st Author	Ref.	Metal	Gas	T, °C	Study
2003	Emmenegger	[12]	Fe/Al	C_2_H_2_	650	Nature of C
2003	Carneiro	[13]	Fe-Cu	CO/H_2_	500–700	Flow reactor
2005	Puretzky	[14]	Fe/Mo	C_2_H_2_	535–900	Mechanism(s)
2006	Karwa	[15]	Steel	Benzene…	725	Self assemble
2007	Masarapu	[16]	Steel 304	Xylene/Ar/H_2_	700	Aligned MWCNT (multi-walled carbon nanotubes)
2007	Zhong	[17]	Fe	CH_4_	600	Mechanism
2007	Iawasaki	[18]	Fe	CH_4_/H_2_	600	Mechanism
2008	Yoshida	[19]	Fe_3_C	C_2_H_2_	600	Mechanism
2008	Baddour	[20]	Steel 304	C_2_H_2_/N_2_	700	Simple procedure
2009	Sengupta	[21]	Fe	Propane/H_2_	650–950	Optimize growth
2010	Li	[22]	Fe, Sn	C_2_H_2_	700	Nanocoils
2011	Nessim	[23]	Fe	C_2_H_4_/H_2_	730–770	Hot-wall reactor
2011	Dasgupta	[24]	Fe,Co,Ni,Cu	Various/CO	550–750	Review (>1996)
2012	Carneiro	[25]	Fe-Ni	CO/H_2_	670	Shape: TEM, XRD
2012	Robertson	[26]	Fe	C_2_H_2_/H_2_	680	CNT (carbon nanotubes) Forests
2013	Hashempour	[27]	Steel	C_2_H_4_	760	Surface treating
2013	Hordy	[28]	Steel	C_2_H_2_	700	H_2_/NH_3_
2013	Patel	[29]	Steel/Fe	C_2_H_2_/CO	800	H_2_/Cr/SS mesh
2014	Zhong	[30]	Fe-Ti	C_2_H_2_/H_2_	700	CNT HD forests
2014	Hashempour	[31]	Steel	N_2_/C_2_H_4_/H_2_	760	Hybrid rate…
2014	Bayer	[32]	Fe/Fe_3_C	C_2_H_2_/NH_3_	750	Fe-C-N solid phase
2015	Gao	[33]	Fe	C_2_H_2_/H_2_/Ar	600	Catalyst lifetime
2015	Romero	[34]	Steel/Fe	Ar, H_2_. C_2_H_4_	716+	VACNTs (vertically aligned CNTs)
2015	Wang	[35]	Steel mesh	C_2_H_4_/H_2_	750	AOB curve layer
2016	Latorre	[36]	Steel foam	N_2_, C_2_H_6_, H_2_	800	Max. 800^0^/FLG
2017	Pakdee	[37]	Steel	C_2_H_2_/H_2_	700~	Amorphous C test
2017	Latorre	[38]	Steel foam	N_2_/C_2_H_6/_H_2_	900	Operation adjust
2018	Thapa	[39]	Steel	C_2_H_2_	650+	Temp.-ramp/NH_3_
2019	Sun	[40]	Steel	C_2_H_2_	760	Substrate surface
2019	Xin	[41]	Steel	CO/H_2_	600	CNTs shape
2019	Roumeli	[42]	Steel	C_7_H_8_/Ferroc.	827	Properties
2019	Panahi	[43]	SS 304,316	Plastic: PE,PP	800	Waste plastics
2020	Hasanzadeh	[44]	Fe,Co,Ni	C_2_H_2_	500+	T: yield + diameter

## Data Availability

Data available upon request.
